# Towards Building a Computer Aided Education System for Special Students Using Wearable Sensor Technologies

**DOI:** 10.3390/s17020317

**Published:** 2017-02-08

**Authors:** Raja Majid Mehmood, Hyo Jong Lee

**Affiliations:** 1 Division of Computer Science and Engineering, Chonbuk National University, Jeonju 54896, Korea; rmeex07@gmail.com; 2 Center for Advanced Image and Information Technology, Chonbuk National University, Jeonju 54896, Korea

**Keywords:** computer aided education, brain signal, EEG based emotion recognition, brain computer interface

## Abstract

Human computer interaction is a growing field in terms of helping people in their daily life to improve their living. Especially, people with some disability may need an interface which is more appropriate and compatible with their needs. Our research is focused on similar kinds of problems, such as students with some mental disorder or mood disruption problems. To improve their learning process, an intelligent emotion recognition system is essential which has an ability to recognize the current emotional state of the brain. Nowadays, in special schools, instructors are commonly use some conventional methods for managing special students for educational purposes. In this paper, we proposed a novel computer aided method for instructors at special schools where they can teach special students with the support of our system using wearable technologies.

## 1. Introduction

In recent years, emotion recognition using brain signals has become more popular due to its low cost with a vast application domain, such as medical applications, electroencephalogram (EEG)-based games, and computer aided systems for students with mental disorders [1,2,3,4]. Human computer interaction has become a part of everyday life. Similarly, emotions are important and constantly exist in a person’s daily life. Emotion-related expression is ubiquitous in the daily routine [5,6,7]. It is an important factor in human interaction and communication. Emotions can provide many possibilities for enhancing the interaction with human subject using the emotion-based intelligent systems, e.g., affective interaction with autism or epilepsy patient [8]. Human learning process is significantly influenced from emotional behavior of subject [2,9,10]. More specifically, while students are attentive with pleasant feelings, they will definitely produce positive results.

In general, teachers observe students’ expressions through face-to-face communication which is extremely difficult in the case of handling the students who have some mental disorder. Therefore, it is required to develop a computer aided education system (CAES) which is capable of detecting the current mood of the subject during his/her class time or pre-school time. It facilitates the teachers in special schools where student have a learning difficulty due to their physiological disorder. To improve the learning process of such students, an intelligent EEG-based emotion recognition can play a vital role [11,12].

Intelligent instructor systems are designed to support instructors in order to improve the learning experience by providing effective treatments to student [13]. Such systems have been found to benefit students by significantly increasing learning through cognitive models [14]. However, many researchers have yet to consider an affective model which has been found to have a great impact on learning process [14]. The use of an affective model in CAES will allow the system to identify emotions and feedback the system accordingly avoiding emotions associated with a decrease in learning such as sadness. In CAES, all emotions are not relevant in order to improve the students learning; only specific emotions that are directly related to learning are known as “academic emotions” [15]. Pekrun et al. [15] developed the academic emotions questionnaire (AEQ) in order to study academic emotions. Their studies show that a wide range of emotions were observed in an academic setting and positive emotions were described just as frequently as negative emotions. Examples of the “academic emotions” included in AEQ were emotions such as happy, scared, sad and calm in arousal-valence domain. For emotion recognition from human brain, it is made possible by using of EEG sensors that can monitor some of the cognitive processes that occur within the brain that relate with certain forms of inner thinking.

State-of-the-art emotion recognition methods have been successful in associating the emotional changes with the EEG signals, and so they can be identified and classified from EEG signals if appropriate stimuli are applied [16,17,18,19,20]. However, automatic recognition is usually restricted to a small number of emotion classes mainly due to signal’s feature and noise, EEG constraints and subject-dependent issues. Accordingly, in this research a novel feature-based emotion recognition model is proposed for EEG-based CAES interface. Unlike other approaches, our research explores a wider set of emotion types, claiming that combining a mutual information-based Hjorth parameters and classification technique may improve the accuracy of the emotion recognition task [21].

Our system mainly aimed to provide an emotion recognition and decision support in case of unpleasant situations of student. It will help the instructor to determine the current situation of the student. The system includes the facility for instructors to create and store treatment instructions against the current emotional conditions of subjects. Instructors may use treatment features when students have any emotion dysregulation, such as unpleasant feelings or boredom, etc. The system keeps all moods-related treatment instructions for each student separately. These treatment instructions will pop up automatically whenever a student is in a similar emotional condition in the future.

The study protocol was approved by the institutional review boards (IRBs) at Chonbuk National University (CBNU-IRB 2013-4). All participants provided IRB-approved written informed consent prior to study participation. The research methodology is explained in Section 2, which includes the proposed system architecture and its implementation. A detailed description of CAES is provided in Section 3. The discussion on our work is described in Section 4. Finally, we presented our conclusions in Section 5.

## 2. Materials and Methods

CAES is software written almost entirely in Matlab scripts and Java. All functions are accessible through a friendly user interface, without any interaction with Matlab, so the CAES library can be used without any Matlab programming experience. Matlab provides a facility to use the EEGLAB [22] functionality for any required processing of brain signals from an Emotiv headset. CAES graphical user interface (GUI) is written in Java with integration of the Emotiv SDK (EDK). The use of EDK provides an interface to the Emotiv headset. In this way, we can connect with the headset for recording EEG signals, channels connectivity, etc. The use of Matlab and Java make CAES a fully portable and cross-platform application.

Electroencephalography is by no means a new technology, being first used to record the electrical activity of a human brain by Hans Berger in 1924. Traditionally, PEMED-MedCon: VG. Nihon Kohden [23,24,25] and Admar Neuro-Cadwell Easy II EEG [26,27] are commonly used EEG devices in many studies. Moreover, there are several options such as TrueScan32 (DeyMed Diagnostics, Payette, ID, USA), ActiveTwo Analog Input Box (BioSemi, Amsterdam, The Netherlands), Easy II EEG PSG Machine (Cadwell, Kennewick, WA, USA), 2EB Clinical System (BrainMaster Technologies, Bedford, OH, USA), and selected TrueScan32 as the most attractive option with respect to their criteria [28]. While researching the suitable options for our target application of consumer grade brain computer interface, we’ve found that affordability, portability and ease of use are not available in any single product.

For practical EEG applications, costs, placement, and connectivity are major factors to be considered as minimal. The advantages of our selected EEG headset are its low price, mobility, and minimum setup time. The EEG signals were recorded with the Emotiv-EPOC System [29]. The sensors are polycarbonate and the device includes 14 electrodes with two reference channels that offer accurate spatial resolution. The device has an internal sampling rate of 2048 Hz before filtering. To minimize the output bandwidth, Emotiv-EPOC passes the 0.2–43 Hz with digital notch filters at 50 Hz and 60 Hz. The normalized EEG signals were obtained through device built-in digital 5th order Sinc-filter. The output sampling rate is 128 samples per second. We selected a 10/20 electrode placement, which is the most effective international standard for capturing reliable EEG recordings with the least number of electrodes [30]. The 10/20 EEG electrode placement is the commonly used standard by most researchers for EEG-based emotion recognition through audio and visual stimuli. This system is based on the relationship of different electrode positions located on the scalp and the primary side of the cerebral cortex [31]. Emotiv headset have a total of 16 EEG channels (AF3, F7, F3, FC5, T7, CMS, P7, O1, O2, P8, DRL, T8, FC6, F4, F8, and AF4), which were inserted for recording EEG signals. Average reference channels (CMS/DRL) were placed in the P3/P4 locations.

**Figure 1:**
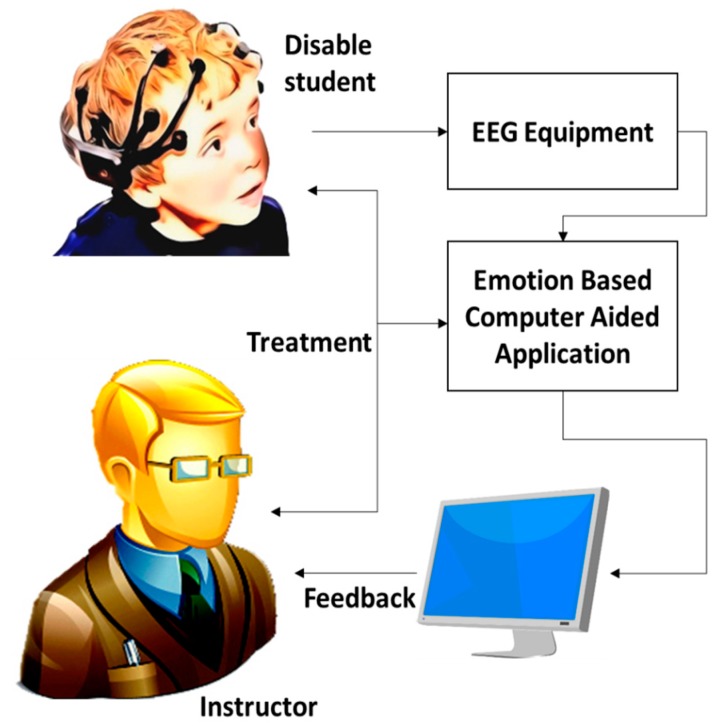
Computer-aided education system.

Figure 1, shows the abstraction level of proposed system design. Students and instructors are the primary users of our proposed system. Instructors have a direct connection with the system and students. Students with some disability need to wear the brain signal device during class period. The device has a wireless interface with application software, so the instructor can see the emotional changes of the student through a feedback channel. At the end, the instructor may suggest and record the treatment procedures in the case of the worsening conditions of the subject. These treatment procedures are going to be stored in a central database and all treatment information is accessible all the time.

Figure 1 shows a direct relationship between instructor and student. In order to improve the learning process of special subjects, the instructor has to monitor their mood status at the beginning of every learning session. The instructor then responds to the students on the basis of their cognitive and affective states. For example, if a student has shown some mood disruption (fearful/sadness), the instructor should need to treat a special subject carefully to improve his/her emotional feelings towards a relaxed or normal state. Further, CAES has a core feature to maintain the complete school database which contains the all students’ treatment plans. This feature will enable the system to provide a full coverage of students’ mood behavior, more effectively.

### 2.1. System Level Design

**Figure 2:**
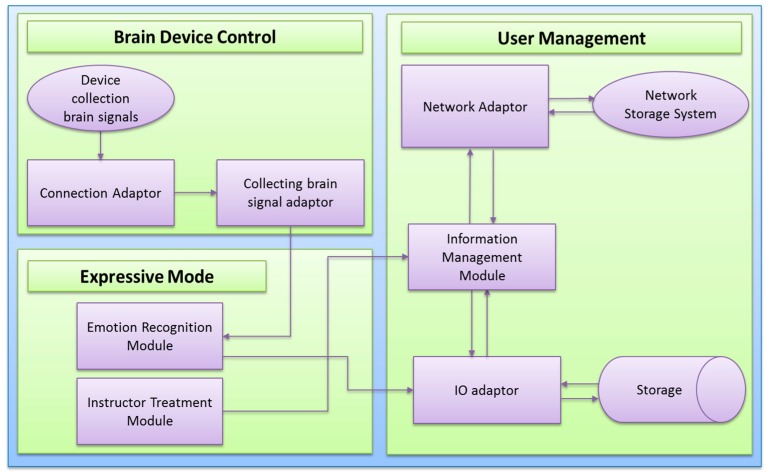
System level component diagram (main system components).

We would like to explain the technology, which is used in the proposed system. Figure 2, shows the data flow of the proposed system. The diagram flow starts from EEG brain device through a Bluetooth connection adaptor (BAC). The connection adaptor provides a connection to the brain signal adaptor (BSA) and it goes directly to the emotion recognition module (ERM). This component includes the emotion detection and other modules which will be discussed in further sections. This component can forward data to the storage devices (SD), network storage system (NSS) and information management module (IMM) through IO adaptor (IOA). IMM is also connected to instructor treatment module (ITM) for retrieval and updates of treatment procedures.

**Figure 3:**
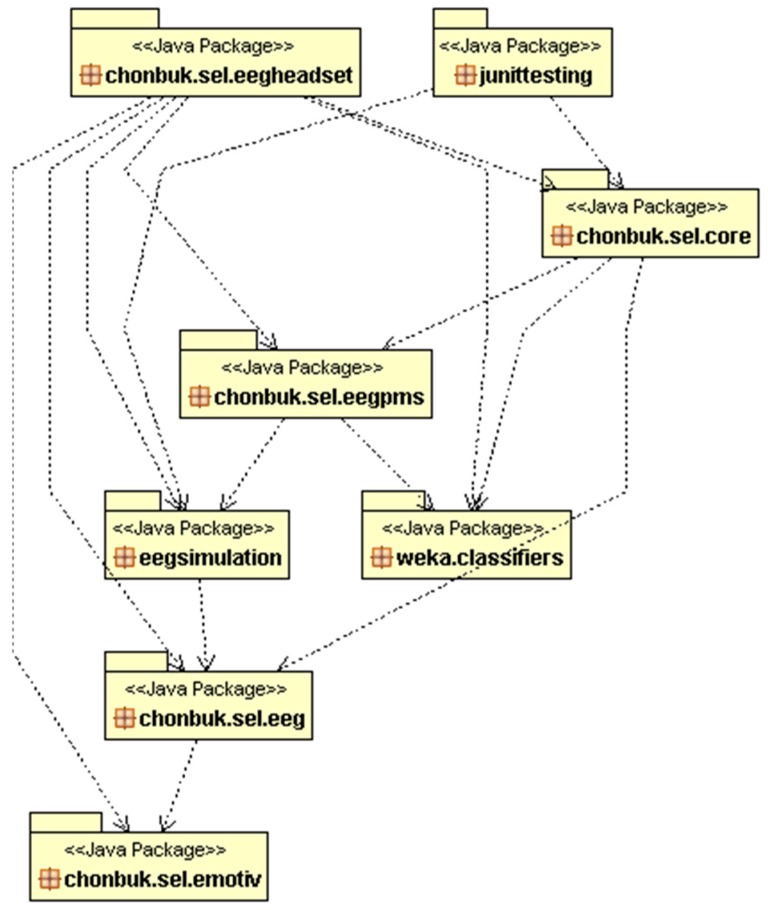
CAES package level diagram, includes most common packages in system development.

In Figure 3, we are presenting the package diagram of our CAES development. It contains several packages which are representing the main components of our system. All of our designed packages are inside ‘chonbuk.sel’ which represents our organizational flow. Each package has its own functionality and purpose, such as:

(1) chonbuk.sel.emotiv: the package named ‘emotiv’ has a main purpose of connectivity with the Emotiv headset using Edk.dll, where Edk is a library for providing an interfacing between EEG machine and our development components. Edk class is bundled with a collection of functions which are easily accessible in our Java program for example, headset signal streaming, EEG channel connection quality and headset battery status.
(2) chonbuk.sel.eeg: this package has been placed between ‘emotiv’ and the other system packages. It has been used for calling the functions of Edk from our programs which is a kind of interfacing. This package contains an EEGDriver class, which is a kind of singleton instance which provides the real-time EEG data to caller programs.
(3) chonbuk.sel.eegheadset: this package is responsible for presentation of real-time EEG data on the screen with other headset’s functions such as battery status, channel connectivity status, etc. It also contains the functionality of managing the EEG data streaming by using of packages such as, ‘eeg’ and ‘emotiv’.
(4) weka.classifiers: this package is an external package from WEKA which contains a bundle of machine learning functionalities such as, SVM, KNN, and so on.
(5) chonbuk.sel.eegsimulation: this package has classes for performing the simulation for training purposes.
(6) chonbuk.sel.eegpms: this package corresponding to user profile management, such as for creating a new user, storing user EEG data into database. We kept the user database in the form of Java object file for each user, separately.
(7) chonbuk.sel.core: this is our core package, where emotion recognition module of our system works perfectly. This package is linked with main functionality of our system such as, computation of Hjorth parameters, EEG band pass filtering, emotion recognition, instructor treatment, etc.
(8) junittesting: at the end, we performed the unit testing of the developed system. The testing was performed only on the main functions of CAES for example; EEG data storage and retrieval, emotion recognition verification, etc.

**Figure 4:**
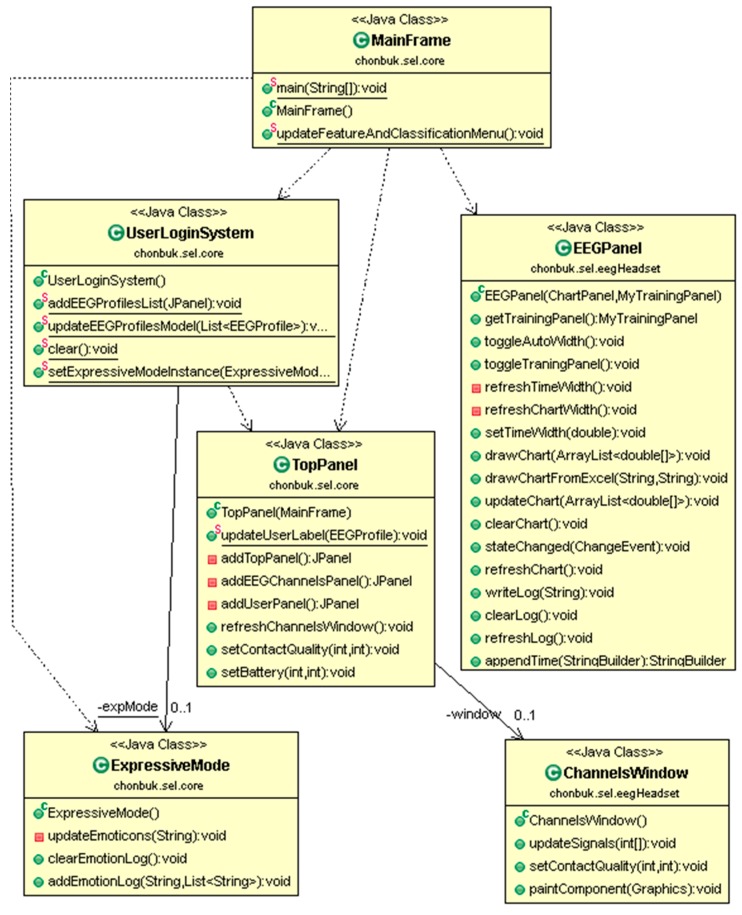
Class diagram representing the main graphical user interfaces of system.

Figure 4 presents the class diagram of our development of graphical user interfaces. It contains the main interface in MainFrame which is a top level interface of our system. UserLoginSystem class has a user login functionality with existing users’ profiles. EEGPanel is part of the ‘eegheadset’ package which corresponds to real time EEG data visualization. TopPanel is a top panel of CAES, where we can see the EEG channel’s connectivity status, logo, and user information. ExpressiveMode class is responsible of emotion recognition module of our system with collaboration of other classes such as classes from WEKA.

**Figure 5:**
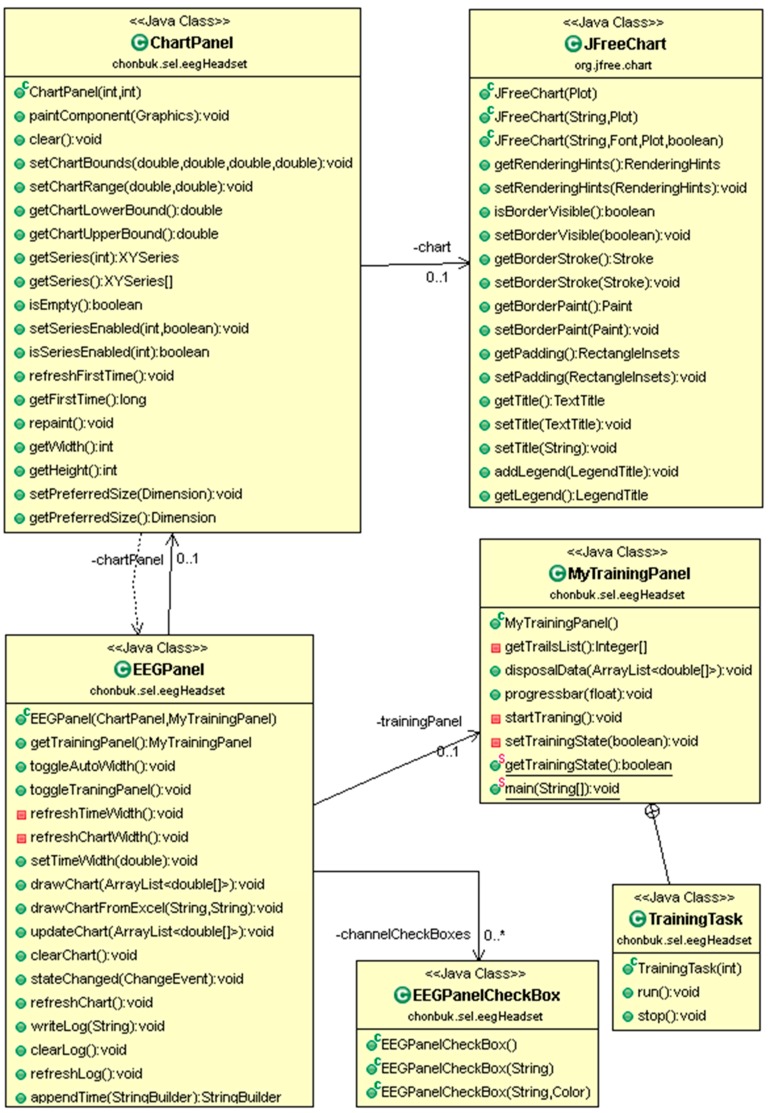
Class diagram for headset package and its functionality for visualizing EEG data and user training management.

The following class diagram in Figure 5 is about the functionality of ‘eegheadset’ which is responsible for EEG data acquisition and real-time EEG visualization. EEGPanel is the top level class of this package which contains the ChartPanel, MyTrainingPanel, and EEGPanelCheckBox. JFreeChart is an external class from an open source chart visualization project and it is embedded through the ChartPanel. TrainingTask is a multithreaded inner class of MyTrainingPanel which has user training session functionality for recording an EEG data for training purposes.

**Figure 6:**
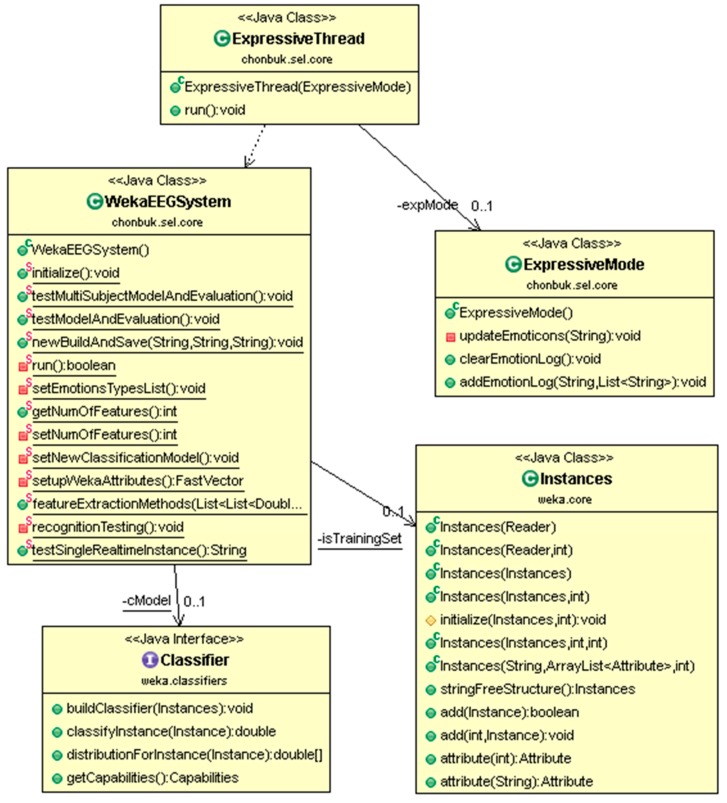
Class diagram contains class structure of emotion recognition module which has linking with WEKA for emotion classification.

A more specific class diagram of emotion recognition using the WEKA interface Classifier is presented in Figure 6. WekaEEGSystem is a top level class is running under ExpressiveThread in its own separate thread instance. The Instances class from WEKA has been used for creating training and testing datasets. Later, these datasets were used by WekaEEGSystem through Classifier. Moreover, ExpressiveThread uses and is dependent on functionality, by instance of ExpressiveMode.

**Figure 7:**
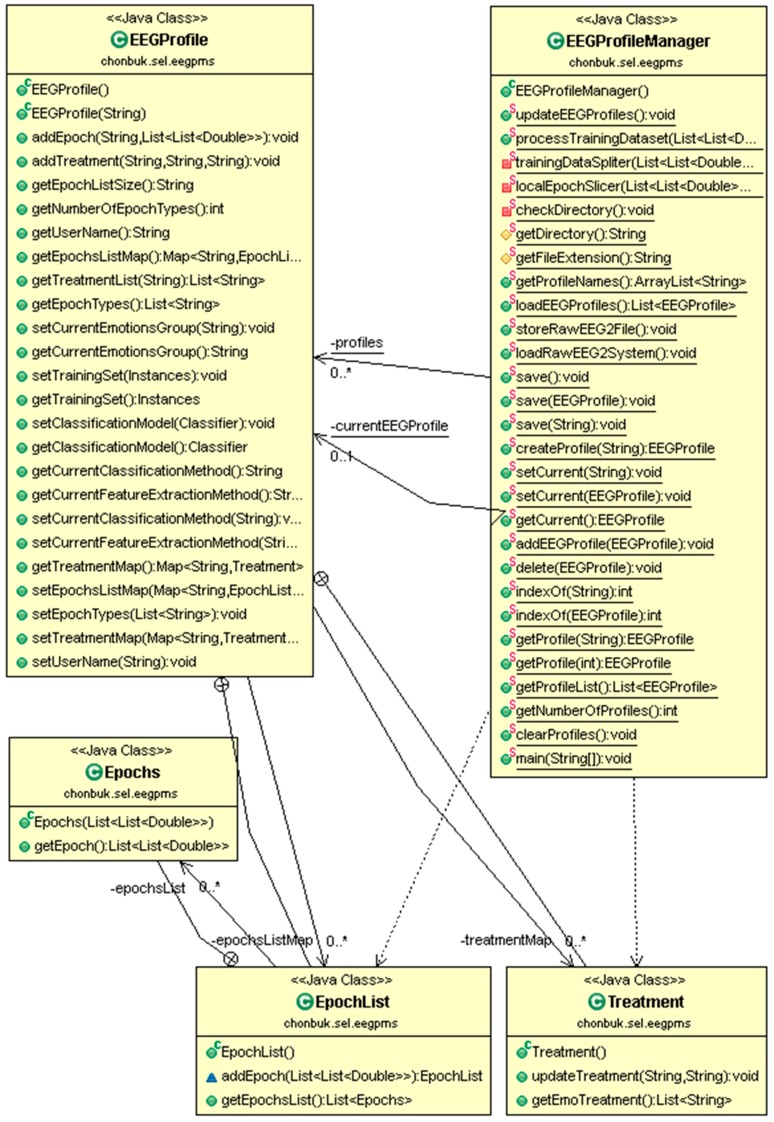
Class diagram for user profile management.

Finally, we present the class diagram for the user profiling system in Figure 7. EEGProfileManager has a lot of facilities to manage the EEG user profile instances. EEGProfile corresponds to each single user profile which contains the user EEG data in the form of training epochs, user naming information, and treatment plans, if any treatment is inserted by any instructor. Treatment and EpochList are inner classes of EEGProfile and Epochs class is an inner class of EpochList. Epochs represent a single EEG signal epoch and is stored in the Epochlist List data structure. There are get methods in all classes which are used to retrieve the stored information of the corresponding user profile.

### 2.2. The Proposed System Methodology

**Figure 8:**
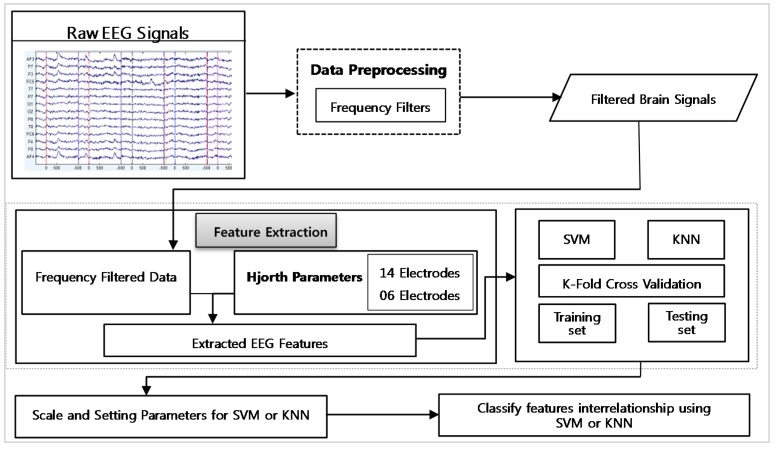
Emotion Recognition Module.

Figure 8 shows a detailed description of ERM. This component takes a single input connection which contains the brain device signals. The data preprocessing unit filters the signals’ data through frequency filters from 0.5 to 30 Hz. It includes four frequency filters such as delta, theta, alpha, and beta. These frequency filters are applied to each brain signal separately. The filtered data is forwarded to the feature extraction module of this component. We employed three kinds of features such as: (1) frequency filtered data; (2) Hjorth parameters with 14 brain signals; and (3) Hjorth parameter with 06 brain signals. The Hjorth parameters from 14 brain channels were selected from all brain regions such as, frontal, central, temporal, parietal, and occipital, but the Hjorth parameters from 06 brain channels were selected from only frontal and central brain lobes. Further, we extract these features from brain signals and process them in the two different classifiers that are support vector machine (SVM) and k-nearest neighbor with 10-fold cross validation. At the end, this component finalizes the current emotional status against the input signals and forwards the current subject emotional status to the IMM component of the system.

The proposed system collects the signals from a brain device (EEG-device) which is located on the scalp of a subject. The signals are processed through a Bluetooth connection adaptor (BAC) that is provided by the Emotiv development kit (EDK) library [29].

**Figure 9:**
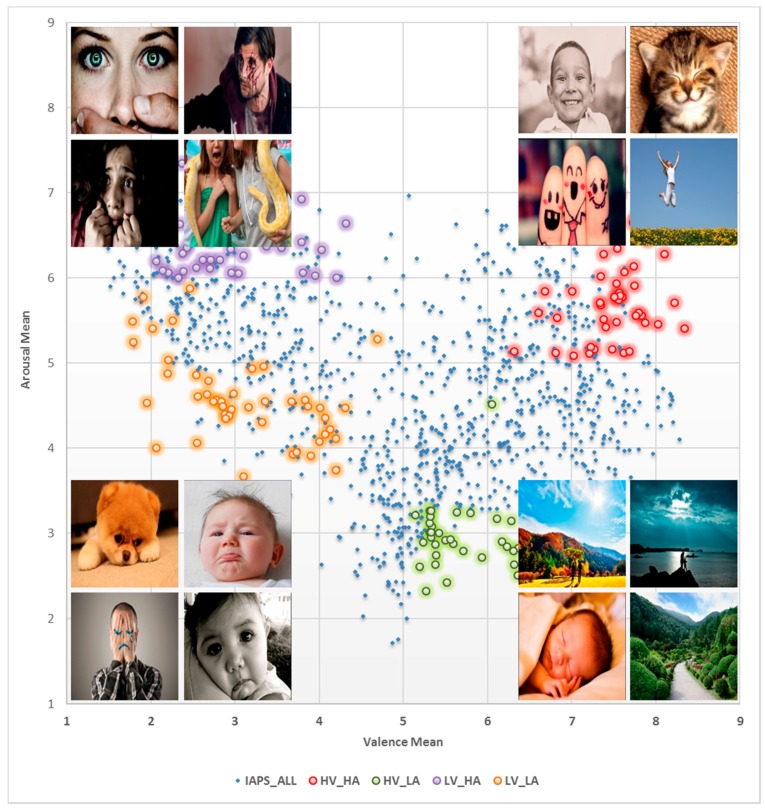
Sample images from the IAPS images dataset selected for training sessions.

The system allows users to record the training sessions of subjects by running an emotion-based simulation. The system has predefined images set of four emotional states: sad, scared, happy, and calm. A total of 180 images (45 pictures × 4 states) was selected from equally distributed groups along the arousal-valence axes of international affective picture system (IAPS) database [32,33,34]. Further, when the training session completes we use the WEKA [35] data mining library to build the emotion classification model. The emotion model will be stored for emotion detection in the expressive mode of the system. Figure 9 shows the IAPS dataset selection for CAES, it also shows the sample emotional images in the corresponding coordinates. The top/bottom-right/left coordinates represent the happy/calm and scared/sad emotion related images in the IAPS dataset. During training sessions, instructors have the option to start training using these images according to system settings.

The expressive mode of the system detects the current mood of subjects and shows the emotional avatar. Here, an instructor may add or update the treatment procedure of subjects according to a subject’s mental condition. The expressive module shows the treatment suggestions to the instructor in the case of an unpleasant situation of a subject, if any treatment guideline is already stored in previous sessions. Furthermore, the following pseudocode explains the systematic flow of the proposed system in a real time environment.

Algorithm 1 is showing the core functionality of CAES. It two conditional statements, one is for ExpressiveMode, and the other one is for TrainingMode. The ExpressiveMode is used for emotion recognition and it will help the instructor manage the students. It actually consists of two main modules that are ERM and ITM. We employed different feature extraction methods in ERM such as the filtered brain signals and Hjorth parameters. The feature vector computed from filtered brain signals contains the maximum length of 2688 data points in each feature vector for each emotion. The second feature extraction method is known as the Hjorth parameter extraction. These parameters are statistical functions which describe the characteristics of brain signals in the time and frequency domains. The Hjorth parameters are also known as normalized slope descriptors (NSDs) that consist of activity, mobility and complexity. The parameters can be computed based on the following derivations.
**Algorithm 1.** CAES in real time settings**Begin**
**Step** **1.**user login to the system**Step** **2.**establishing a device connection using EDK**Step** **3.****Repeat****Step** **4.** acquiring brain signals**Step** **5.** user has to select either ExpressiveMode or TrainingMode**Step** **6.** if ExpressiveMode equals to true**Step** **7.**   extract raw signals**Step** **8.**   applying frequency filters**Step** **9.**   extract Hjorth features from all signals**Step** **10.**  calling classification module for emotion detection**Step** **11.**  rendering the emotions avatar**Step** **12.**  displaying instructions of treatment according to subject condition**Step** **13.** end**Step** **14.** if TrainingMode equals to true**Step** **15.**  start emotion based simulation**Step** **16.**  recording of brain signals**Step** **17.**  if Simulation equals to end**Step** **18.**   building a classification model using WEKA library**Step** **19.**   store classification model into database**Step** **20.**  endif**Step** **21.** endif**Step** **22.****until** stop**End**


The instructor has to set up a training session with a student in the case of TrainingMode which is activated by him/her. The instructor will record the brain signals from students under his supervision. Training sessions are more sophisticated and important for correct emotion recognition. Therefore, the instructor should monitor the students’ physical behavior during the training session. He/she can also guide the student about the training session before the session starts.

The automated program will process the brain signal recordings after the completion of every training session. The system process also includes the band pass filtering and building the new classification model. The proposed system allows users to train multiple sessions at any time. Every time the system will update the existing training model with new recorded brain signals.

## 3. Computer Aided Education System (CAES)

However, the existing methods are extremely subjective and waste a significant amount of energy of the teachers while instructing special students. The proposed system CAES helps the instructors or teachers in special schools where they have to teach disabled students. The CAES works with the brain device which measures the electrical signals from a human scalp in real-time. This system is aimed at helping instructors deal with students in special schools. These students may have some kind of physical disabilities, for example, autism or epilepsy, etc. The instructor has to operate this system with target students, which helps them recognize the current emotional condition of subjects.

**Figure 10:**
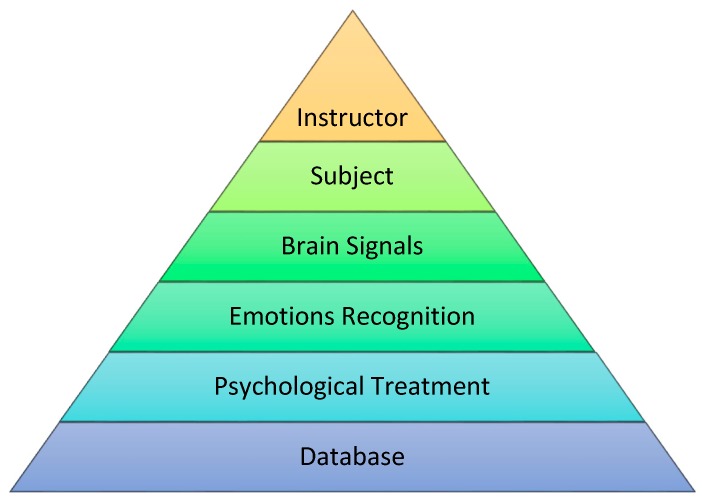
CAES Process Flow Diagram.

This system has proposed a novel approach to help instructors at special schools, whereby instructors may improve the learning process of disabled students. The proposed method includes the emotion recognition based on brain signals and intelligent decision support for instructor in case of some unpleasant condition of a student. Figure 10 gives an overview of the system flow of CAES.

- The instructor is the system admin and it helps the subject in the whole process.
- The subject is an end user of the system. Its inner brain activity generates a signal pattern.
- Brain signals are collected from the scalp of subjects.
- These emotions (happy, calm, sad, and scared) are selected for recognition.
- The psychological treatment always depends on the behavior or emotion of each subject.
  ○ The system allows the instructor to add or update the treatment plan of current Subject.
- The database is the central repository of all treatments and procedures of subjects, which are recorded by instructors.

The proposed system is bundled with main features such as user management, brain device control panel, expressive panel, treatment panel, etc. The following features are the main components of our proposed system.

### 3.1. List of Features

- User (Student) Information Module
  ○ It contains the existing user of the system
  ○ It allows to create new User or Subject
  ○ It allows the User to connect to brain device
- Device Control Module (Headset Setup)
  ○ It shows the current status of all device electrodes
  ○ It shows the signal activity in brain through each electrode in 2d Chart
  ○ It shows the battery status of the device
- Events Module
  ○ This module records all activities of system such as, device connection, exceptions, training, user login, logoff, etc.
- Expressive Module
  ○ It shows the current mood of the subject
  ○ It records current mood of the subject
  ○ It allows the instructor to add or update new treatment of current subject
  ○ It guides the instructor about the treatment plan for current subject

### 3.2. System Major Components and Functionalities

**Figure 11:**
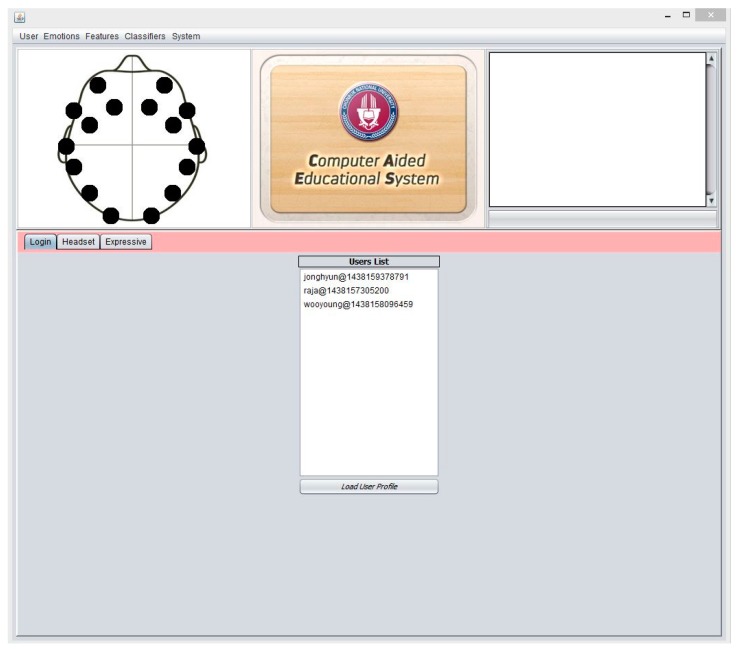
CAES (Main GUI) shows the main interface of the proposed system; it includes menu options for the end user to access different functionalities of the system; the login window provides an interface for users to enter the system, headset setup includes the device-related functions and the user training module; expressive mode is a core component of our system which contains the emotion recognition and treatment instructions for instructors.

**Figure 12:**
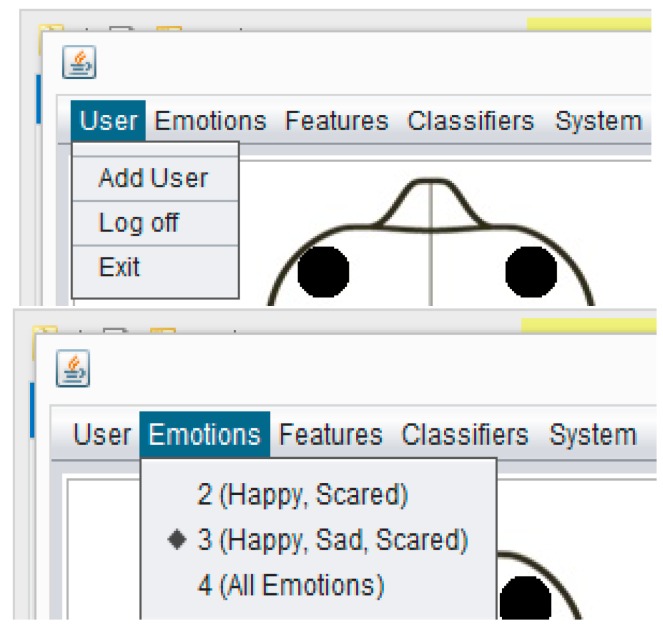

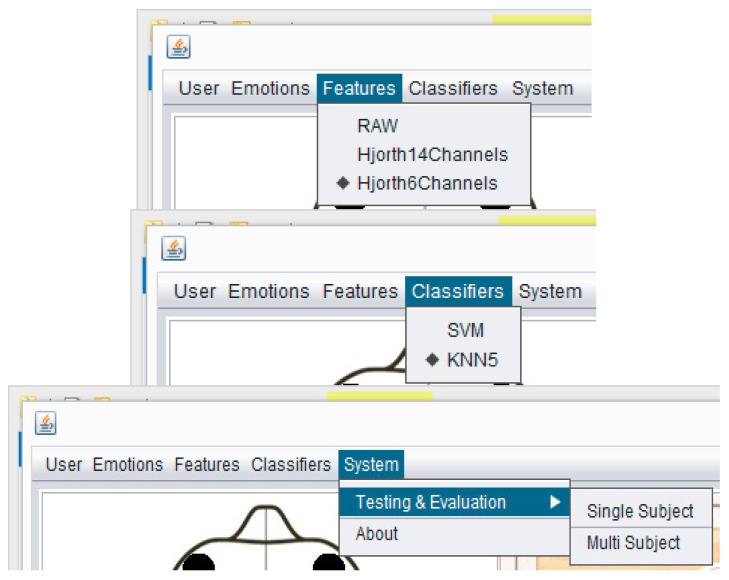
CAES (menu details) shows the menu options of the proposed system. A user can select any option from the above menu and he/she will see the results according to his/her menu-option selection. Different kinds of functions are accessible through the above menu options depending on the user requirements; for example, if the instructor is only interested in two emotions (happy, scared) he needs to choose the first option in the emotions menu, and similarly users have different features and classification options.

**Figure 13:**
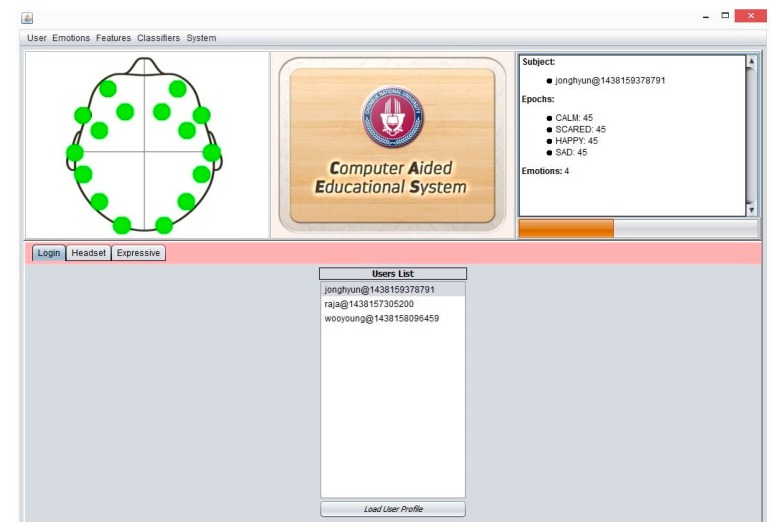
CAES (User Login); it provides the facility for users logging into the system. At this point the user can see the signal quality of the Emotiv headset channels, and the user can see the current user details such as name, epochs, etc.

**Figure 14:**
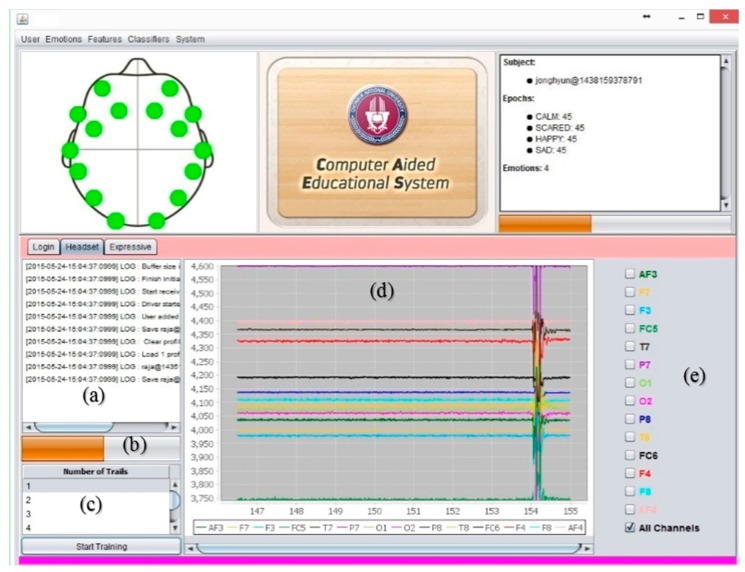
CAES (headset setup); this is also known as the device control module: (**a**) this component shows the device-related events and records into the database; (**b**) is the training progress bar; (**c**) the training component that allows the instructor to record the emotion training of the current subject; this module computes and updates the classification model after each training session; (**d**) this component shows the signal activity of each electrode of the brain device; (**e**) this component helps the user select his/her desired brain electrode.

**Figure 15:**
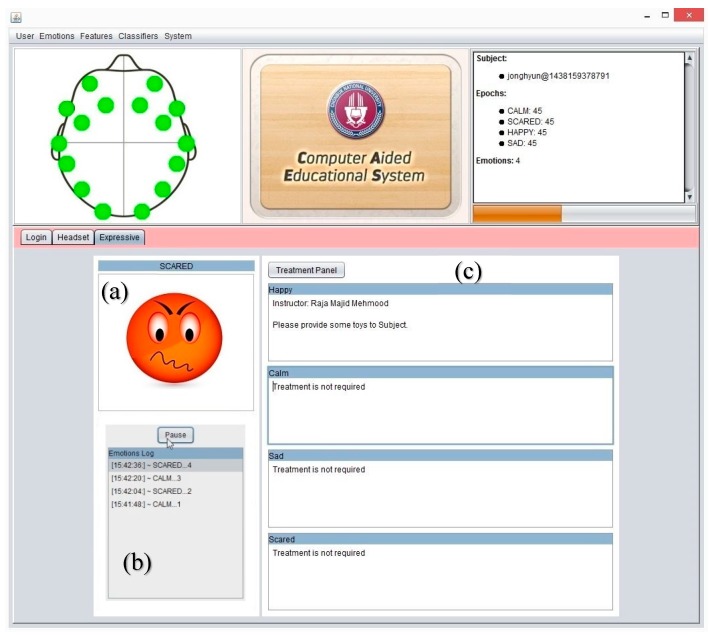
CAES (expressive mode), is a core component of our proposed system: (**a**) it displays the emotional avatar against the current mental condition of the subject; (**b**) this contains the list of emotions and helps the instructor see the subject’s previous behavior, especially in unattended situations; (**c**) this component guides the instructor with detailed suggestions from other instructors of the same subject. In this way, any instructor can easily understand the subject conditions and is also helpful for him to deal with the current situation.

**Figure 16:**
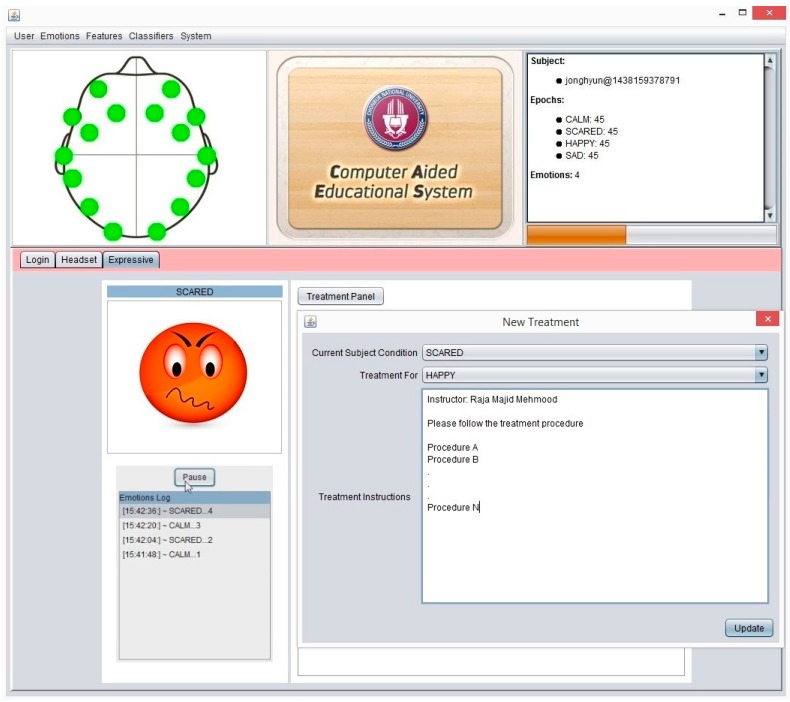
CAES (New Treatment): the instructor is allowed to add or update the new treatment procedure for current emotional state.

CAES is used through its interface which has a variety of functions. These complex functions are easily accessible on the top level GUI which has been added to simplify access by the end users. This system is itself a generic environment structured around one unique interface in which specific functions were implemented (Figure 11). From the user perspective, its component structure is more contextual rather than linear: the multiple features of the software are listed in simple menus; some other specific functions are accessible only when needed and are typically suggested within contextual tab windows. In this way, the system provides a user-friendly access to user in order to provides faster and easier access to the desired functions. In Figure 11, the main system’s GUI provides access to end users. Three main components of the system are displayed: (1) menu section, including a variety of functions; (2) top panel: showing a headset channel, system logo, and user information; (3) tabbed panel: including the user-login, headset-mode, expressive-mode. Further, the CAES main components will be explained through the following system level interfaces from Figure 11, Figure 12, Figure 13, Figure 14, Figure 15 and Figure 16.

## 4. Discussion

It is well known that emotions play a vital role in our daily life. Emotional behavior has a crucial involvement in students’ studies. More specifically, while students are attentive with pleasant feelings, they will definitely produce positive results. Therefore, we have proposed a system (CAES) which has the ability to detect the current mood of subjects during his/her class time or pre-school time. It helps the teachers in special schools where students have learning difficulties due to their physiological disorder. The proposed system is designed to support instructors in order to improve the learning experience by providing effective treatment for students.

This paper describes a technology used in intelligent CAES to recognize the emotional activity and respond to student affect in order to improve his/her learning capabilities. We identified emotion indicators in valence and arousal states linked to student learning, as well as physical behaviors linked to emotional states. The proposed system gives feedback to instructors which guides further treatment in case of any mood disruption in students. As mentioned in Figure 16, we can see the treatment guidelines are available for all instructors in each targeted emotion module. The instructor-on-duty may use the previous mood history and treatment guidelines of current subjects in case of any mood disruption problem. Instructors also can modify any part of the emotion treatment guidelines, per the requirement.

In this study, we consider that instructors are well trained in handling the students in special schools. To improve the learning process of students, instructors should follow the special mood treatment procedures for each individual, separate. Once an instructor is well aware of student’s current mood, he/she can teach him/her accordingly. There is also the possibility that some students with severe symptoms may require home instruction, hospitalization, a self-contained program, day treatment, or a residential setting.

This study adopted the IAPS protocol, while using CAES in order to determine its accuracy and applicability in a school environment. To identify the correct emotional activity of subjects, it is very important to train the subjects properly for different emotional changes. Academic emotions in arousal-valence were included in the system for training purposes. To stimulate the emotional activity inside the brain, IAPS images were integrated in the system which can be used during subjects’ training sessions. The system is capable of doing multiple training sessions anytime, which will help record subjects’ mood changes frequently, therefore we developed a system with emotional-feedback for subjects’ treatment in case of emotional disruption or dysregulation. All treatments are well synchronized and recorded under the current user’s login. Later, these treatment records will pop up in case of any emotional changes in a subject while wearing the EEG headset.

The system was tested on an Intel Core i5 machine with 8 Gigabytes of internal memory. Emotional feedback accuracy was tested using the emotion recognition method described in [21] which is adequate enough to deal with subject mood problems. Due to limitations in accuracy using the EEG headset, we preferred to recognize ten emotions from subjects over 15 s of time. Later, the final single selected emotional-feedback is generated based on the majority in the emotional classes. In this way, we could minimize the error rate in order to improve the student’s learning process. Overall, our developed system is based on inexpensive EEG-devices which are easily available on the market. The EEG-device is a lightweight, easy to set up, and comparatively cheaper consumer-grade EEG device. The complete software application is bundled with a single executable file which is also very lightweight in processing.

Unit testing was performed to verify the system quality and performance. We had used the junit testing framework in Java for unit testing at the module level. In this way, we tested the core public functions of CAES. Further, we also performed a heuristic evaluation of CAES whereby tests were performed on five subjects requiring special assistance. The main purpose of this test is to evaluate the system performance and verification.

Heuristic evaluation is a methodical procedure to check user interfaces for usability problems. Once a usability problem is detected in a design, they are attended as an integral part of the constant design processes. Heuristic evaluation methods include some usability principles such as Nielsen’s ten usability principles, so we considered the following rules which were proposed by Nielsen.

In the above Table 1, ten principles of Nielsen serve as a checklist in evaluating and explaining problems for the heuristic evaluator while auditing an interface or a product like the HCI system.

**Figure 17:**
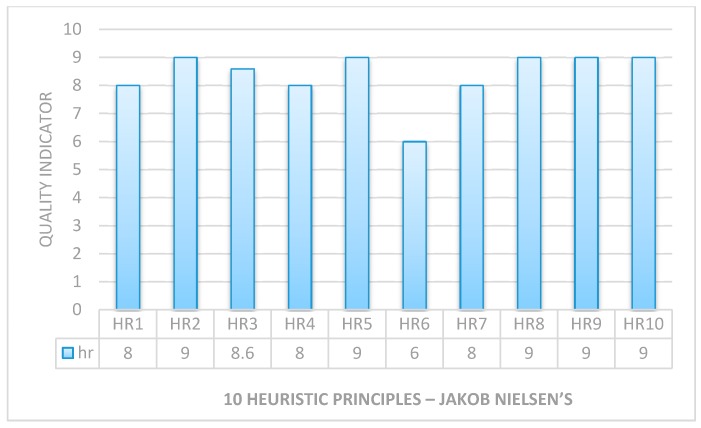
System usability validation using heuristic principles.

For testing purposes, we graded each of the above rules between 0 and 9 for every heuristic test (five times). Low system quality was graded as zero (0) and high system quality was graded as nine (9). Later, we estimated the overall performance of the system by simply averaging the results from each test against each rule and the results are presented in Figure 17. Only HR6 shows a lowest rating, which is obvious because there is no auto text prediction in the case of treatment. The overall heuristic results show the successful test of system usability of our CAES development.

Moreover, we studied several intelligent systems for educational learning and improvement [13,36,37]. Recently, a few studies have described pilot studies on modern teaching aids in education using EEG-based subject’s emotional responses. Their main focus on how to improve the student’s learning process using EEG human brain signals. Researchers also investigated the computational thinking using EEG to improve their learning process and reduce their cognitive workload [38,39], but to-date we could not find any EEG-based computer aided education system of a similar nature.

## 5. Conclusions

The selected EEG-device headset certainly has several advantages over traditional EEG systems, being less expensive, convenient and easy to access. The EEG headset is bundled with a variety of sample programs in EDK which is accessible and customizable in any programming environment. CAES is fully developed and functioning using EDK to send the emotional feedback to the instructor. While investigating the CAES, our main focus was to determine whether the emotional responses of the system and subject matched properly in order to improve the student learning process. Modern EEG-based computer aided systems may help develop the students’ abilities by boosting learning, thinking, communication skills and cooperation. In order to bring subjects to a stable learning state, instructors may have to instruct the subject in relaxation and environmental changes. Relaxation exercises include deep breathing, slowly repeating calming phrases, use of imagery, and trying some non-strenuous or slow exercises. As for environmental change, it can be some break time, video games and visual simulations. Moreover, CAES represents a very important contribution towards academic and practical research, and intelligent CAES has the ability to recognize the emotional activity and respond to students’ emotions in order to improve his/her learning capabilities. In this system, emotion indicators in valence and arousal states are linked to student learning, as well as physical behaviors linked to emotional states. The proposed system gives feedback to instructors which guides further treatment in case of any mood disruption in students. The instructor-on-duty may use the previous mood history and treatment guidelines of current subjects in case of any mood disruption problem. This system adopted the IAPS protocol based on academic emotions for appropriate decision making and treatment by instructors.

## Figures and Tables

**Table 1:** Nielsen’s Ten Heuristic Principles.

HR1	Visibility of System Status
HR2	Match between system and real world
HR3	User control and freedom
HR4	Consistency and standards
HR5	Error prevention
HR6	Recognition rather than Recall
HR7	Flexibility and efficiency of use
HR8	Aesthetic and minimalist design
HR9	Help, diagnosis and recovery from errors
HR10	Documentation and Help
